# Evaluating a candidate predictor beyond statistical significance: Atmospheric pressure and psychiatric day-care attendance as a worked example

**DOI:** 10.1371/journal.pone.0352385

**Published:** 2026-11-06

**Authors:** Takahiro Okuma, Minoru Ishige, Shuichi Kikuchi

**Affiliations:** Department of Psychiatry, Sodegaura Satsukidai Hospital, Sodegaura, Chiba, Japan; The Chinese University of Hong Kong, HONG KONG

## Abstract

In observational research, statistical detectability, robustness to adjustment, and predictive utility are distinct properties of a candidate predictor, yet they are often conflated. A statistically significant association may weaken after accounting for co-varying factors and may add little to out-of-sample prediction. We illustrate this distinction using atmospheric pressure and daily attendance at a Japanese psychiatric day-care as a worked example. Using four consecutive fiscal years of facility-level daily attendance, we evaluated same-day atmospheric pressure as a pre-specified candidate predictor in three stages: statistical detectability, robustness to adjustment for co-varying meteorological factors and seasonality, and incremental out-of-sample predictive utility beyond institutional scheduling variables and recent attendance history. Same-day pressure was positively associated with attendance in all four years (incidence rate ratios 1.003–1.007 per hPa). However, effect-estimate stability varied across years, with marked attenuation in FY2022, moderate attenuation after precipitation adjustment in FY2023, and specification-sensitive precision in FY2024, whereas FY2025 was comparatively stable. In contrast, the structural baseline incorporating scheduling variables and recent attendance history provided strong out-of-sample prediction, explaining 55%–69% of the variance across years. Adding atmospheric pressure produced only small and unstable changes in the primary expanding-window cross-validated performance (mean ΔR^2^ ≈ +0.005), with increments varying in sign across years. Thus, a candidate predictor that was repeatedly statistically detectable showed limited stability of effect magnitude or precision under reasonable adjustment and little incremental predictive utility beyond a strong structural baseline. In this highly schedule-driven, single-facility setting, the weather signal examined contributed little incremental information for out-of-sample prediction of aggregate attendance. More broadly, this worked example illustrates why candidate predictors should be evaluated separately for detectability, robustness of effect estimates, and out-of-sample predictive utility before statistically significant associations are interpreted as meaningful or actionable.

## Introduction

In observational research, a statistically detectable association is often treated as evidence that a candidate predictor represents a meaningful and potentially useful signal. These are different claims. Statistical detectability refers here to whether an association meets a prespecified inferential threshold under a specified model; robustness asks whether the direction, magnitude, and precision of that association remain reasonably stable after accounting for plausible co-varying factors and across reasonable model specifications; and predictive utility asks whether the candidate predictor improves performance on observations not used for model fitting. A predictor may satisfy the first criterion without satisfying the second or third. Explicitly separating these properties is therefore important when observational associations are interpreted scientifically or considered for practical prediction [1].

Weather–health research provides a useful setting in which to examine this distinction. Routinely collected meteorological and health-service data make environmental associations relatively easy to quantify, while environmental variables are often correlated with one another and with seasonal patterns. Psychiatric service use is particularly instructive because previous studies have reported associations with temperature, humidity, atmospheric pressure, and other meteorological conditions, predominantly in acute or symptom-driven settings [2–15]. Scheduled psychiatric day-care differs from these settings because attendance is constrained in advance by institutional routine. A candidate environmental predictor must therefore add information beyond a potentially strong structural baseline.

Atmospheric pressure provides a concrete worked example for evaluating this problem. It has repeatedly been examined in relation to psychiatric outcomes, but reported associations vary in direction and magnitude and a specific causal mechanism remains unestablished [11,12]. We specified same-day atmospheric pressure as the single candidate predictor before modeling. Temperature, humidity, and precipitation were not treated as competing exposures; rather, they were used as co-varying meteorological factors in pre-planned robustness analyses. This distinction is important because the purpose of the study was not to select the best weather variable, but to subject one pre-specified candidate predictor to sequential evaluation for detectability, robustness, and predictive utility.

The present Japanese psychiatric day-care provides a particularly stringent setting for this evaluation. Psychiatric day-care is a long-standing component of community mental health care in Japan [16,17], and continuity and engagement in psychiatric services are clinically relevant [18,19]. Registered patients attended on pre-assigned weekdays within a biweekly program rotation, so much of the day-to-day variation in attendance was expected to be determined by institutional scheduling and recent attendance history. In such a structure-dominated setting, any environmental contribution must compete with a strong baseline. Accordingly, we analyzed four consecutive fiscal years of daily attendance and addressed three questions: (1) was the association between same-day atmospheric pressure and attendance statistically detectable after accounting for the scheduling structure; (2) were the direction, magnitude, and precision of that association reasonably stable after adjustment for co-varying meteorological factors, seasonality, and alternative reasonable specifications; and (3) did atmospheric pressure provide incremental out-of-sample predictive utility beyond scheduling variables and recent attendance history? Our aim was not to establish a general biometeorological effect of atmospheric pressure, but to use a concrete observational example to illustrate why detectability, robustness, and predictive utility should be evaluated separately before a candidate predictor is interpreted as meaningful or actionable.

## Related work

### Meteorological factors and psychiatric service use

Meteorological conditions have been examined in relation to a range of psychiatric outcomes, particularly hospital admissions and emergency presentations [2–15,20]. Temperature has received substantial attention, whereas evidence for atmospheric pressure, humidity, precipitation, and other meteorological factors is more limited and inconsistent [11–15]. Studies have reported associations between atmospheric pressure and psychiatric emergency visits or admissions [2–7,14], but their direction and magnitude have varied, and a specific physiological mechanism linking pressure to psychiatric service use has not been established [11,12].

An important feature of this literature is its setting. Most studies concern inpatient admissions or emergency presentations [14,15], where service use is driven at least partly by acute symptom variation. Scheduled community services represent a different behavioral context because the opportunity and expectation to attend may already be strongly determined by institutional arrangements. Evidence from acute psychiatric services therefore cannot by itself establish whether a meteorological variable contributes meaningful information to attendance in a highly structured service. More generally, finding an association between a meteorological variable and service use does not determine whether the association is independent of co-varying weather or whether the variable improves prediction beyond established structural determinants.

### Calendar and weather variables in health-service forecasting

A related literature has treated health-service utilization as a forecasting problem rather than primarily as an exposure–outcome association. Forecasting studies of emergency and outpatient demand have incorporated recent utilization, calendar structure, meteorological variables, or combinations of these inputs [21–25]. Calendar effects, particularly day of the week, holidays, and seasonal patterns, recur as important predictors of service demand [23–25].

Several studies have directly examined whether meteorological information improves forecasting beyond such structural variables. Marcilio et al. compared models for daily emergency-department visits using calendar variables with and without ambient temperature and found that adding temperature did not improve forecasting accuracy [24]. Erkamp et al. compared calendar-only and calendar-plus-weather models using a separate test period and found almost identical predictive performance [25]. Thus, a meteorological variable may be associated with service utilization without providing substantial incremental forecasting value once calendar structure has been modeled. This distinction is directly relevant to scheduled psychiatric day-care, where the institutional schedule may account for a particularly large proportion of predictable variation.

### From association to robustness and predictive utility

The distinction between explanatory association and prediction is well established. Shmueli argued that explanatory and predictive modeling pursue different goals and should be evaluated using criteria appropriate to those goals [1]. Schooling and Jones similarly emphasized that treating a variable as an explanatory factor and treating it as a predictor correspond to different research questions and require different approaches to modeling and interpretation [26]. In prediction research, the value of a new candidate predictor is therefore not established by the statistical significance of its coefficient alone. Its contribution should be evaluated relative to information already available in a baseline model, preferably using performance on data not used for model fitting [27].

A separate issue is whether a detectable association retains a similar interpretation after plausible co-varying factors and reasonable alternative specifications are considered. In observational environmental data, meteorological variables and seasonal patterns are frequently correlated, so a coefficient that is statistically detectable in a restricted model may attenuate or become unstable when related conditions are incorporated. Such sensitivity does not, by itself, identify the reason for the attenuation—confounding, collinearity, measurement error, and sampling variation may all contribute—but it limits the strength with which the original association can be interpreted as an independent signal.

The present study combines these considerations into a three-part evaluation of one pre-specified candidate predictor. We distinguish statistical detectability, robustness to adjustment and reasonable model specification, and incremental out-of-sample predictive utility. Atmospheric pressure serves as the worked example rather than as a claim that pressure is uniquely important among meteorological variables. The same observed association can therefore be asked three different questions: can it be detected, does its interpretation persist under reasonable stress tests, and does it add useful predictive information beyond what is already known?

## Materials and methods

### Study design and setting

This retrospective, facility-level observational study was conducted at the psychiatric day-care unit of Sodegaura Satsukidai Hospital, a general hospital in Sodegaura, Chiba Prefecture, Japan. The unit operated six days per week (Monday–Saturday) with a biweekly program rotation in which the same program content recurred on the same weekday every two weeks. Registered patients attended on pre-assigned weekdays. Patients traveled to the unit on foot, by public transport, or by private car; there was no shuttle service, and the proportion traveling by private car was relatively high compared with typical urban facilities.

The analysis covered four consecutive Japanese fiscal years (FY2022–FY2025), each running from April 1 to March 31 of the following year. Reporting followed the STROBE statement [28].

The study was approved by the Institutional Review Board of Sodegaura Satsukidai Hospital (approval number 2025−12; approval date: April 13, 2026). The present analysis of weather and attendance was conducted as one component of the approved protocol, entitled “An example of applying machine learning to program development in a psychiatric day-care.” Informed consent was waived by the Institutional Review Board because of the retrospective, non-interventional design, and an opt-out procedure was adopted. Attendance records were accessed for research purposes on April 20, 2026. The data used were anonymized, facility-level aggregate counts, and the authors did not have access to information that could identify individual participants during or after data collection. All procedures were performed in accordance with the Declaration of Helsinki and the Japanese Ministry of Health, Labour and Welfare’s Ethical Guidelines for Medical and Health Research Involving Human Subjects (revised 2021).

### Participants and outcome

Registered patients attended on pre-assigned fixed weekdays. Demographic characteristics for FY2025 are summarized in Table 1. The composition of registered patients and weekday-specific enrollment varied across fiscal years.

**Table 1:** Baseline characteristics (FY2025 registered patients; FY2022–FY2025 operating-day statistics).

Characteristic	Value
Participant demographics (FY2025, N = 59)	
Sex, male / female, n (%)	33 (55.9%) / 26 (44.1%)
Age, years, mean ± SD (range)	54.2 ± 13.5 (23–85)
Schizophrenia, n (%)	21 (35.6%)
Mood disorders, n (%)	17 (28.8%)
Alcohol use disorder, n (%)	10 (16.9%)
Other psychiatric disorders, n (%)	11 (18.6%)
Operating-day statistics (FY2022 / 2023 / 2024 / 2025)	
Analytic operating days	266 / 264 / 263 / 262
Mean daily attendance, persons (SD)	17.6 (4.6) / 15.7 (4.3) / 17.8 (4.3) / 14.7 (4.2)
Mean atmospheric pressure, hPa (SD)	1014.6 (6.7) / 1013.9 (6.3) / 1012.9 (6.5) / 1014.4 (6.4)

The outcome was the daily attendance count, defined as the number of patients attending on each operating day. Attendance records were complete for all operating days. Because the outcome was a non-negative count and exhibited systematic underdispersion associated with the fixed scheduling structure, quasi-Poisson regression was used for the primary inferential models.

### Meteorological data and candidate predictor

Daily meteorological data were obtained from the Japan Meteorological Agency and included mean atmospheric pressure (hPa), mean temperature (°C), relative humidity (%), and precipitation (mm). Mean atmospheric pressure and relative humidity were obtained from the Chiba Local Meteorological Observatory, whereas mean temperature and precipitation were obtained from the Kisarazu observation station, both in Chiba Prefecture. Kisarazu is an AMeDAS station and does not measure atmospheric pressure. Relative humidity became available at Kisarazu only from November 16, 2022; therefore, humidity from the Chiba observatory was used throughout the four-year period to maintain a consistent series. The Chiba observatory is approximately 20 km from the facility.

Same-day atmospheric pressure was specified before modeling as the single candidate predictor for the three-stage evaluation described below. Same-day exposure was chosen because weather conditions on the attendance day were considered most directly relevant to same-day attendance. The pre-specified exposure was the same-day mean atmospheric-pressure level rather than within-day or day-to-day pressure change. Accordingly, the present analysis does not directly evaluate possible effects of transient barometric changes. Temperature, relative humidity, and precipitation were not treated as competing candidate predictors. Instead, they were used as pre-planned co-varying meteorological factors in robustness analyses. No data-driven selection among meteorological variables or pressure lags was performed. The study was exploratory and not pre-registered, but the candidate predictor and covariate set were fixed during planning and were not revised after inspection of the results.

### Statistical evaluation framework

The same candidate predictor was evaluated through three distinct questions: (1) statistical detectability, (2) robustness of the estimated association across reasonable adjustments and alternative specifications, and (3) incremental out-of-sample predictive utility. This sequence was used to distinguish evidence that an association met the prespecified inferential threshold from evidence that its direction, magnitude, and precision remained reasonably stable under adjustment or that it improved prediction beyond the structural baseline. Fig 1 summarizes the three stages, the question each addresses, and what each stage does not establish.

**Fig 1:**
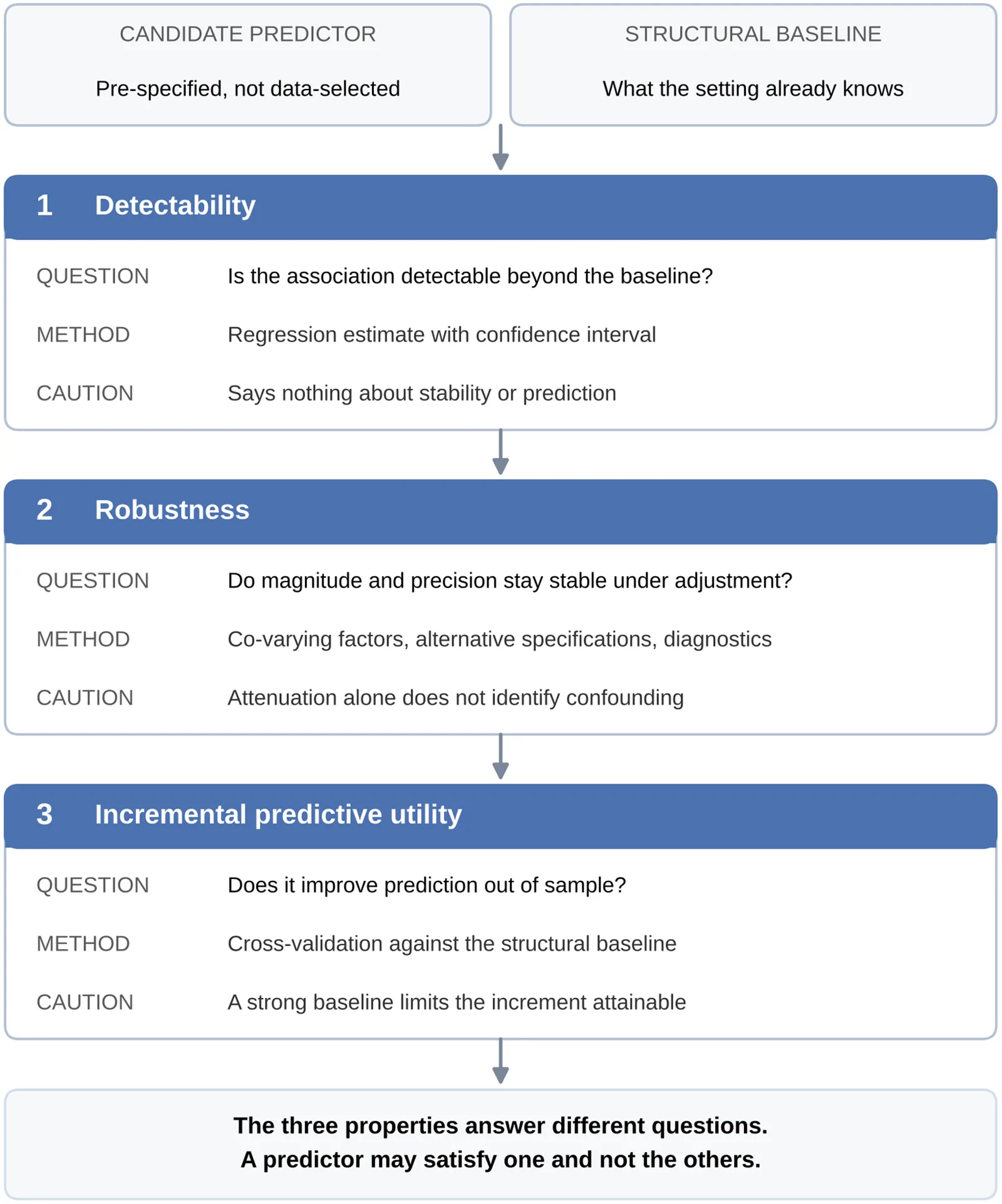
Framework for evaluating a candidate predictor. A pre-specified candidate predictor is evaluated in three stages against a structural baseline formed from information the setting already holds. Each stage answers a different question, so satisfying one stage does not establish the others. In the present study the candidate predictor was same-day atmospheric pressure and the structural baseline comprised weekday indicators and attendance 14 days earlier; the three stages correspond to Table 2, Tables 3 and 4, and Table 5, respectively.

#### Detectability.

For each fiscal year, daily attendance was modeled using a quasi-Poisson generalized linear model [29]. Quasi-Poisson estimation was used to accommodate underdispersion induced by the fixed scheduling structure while retaining inference on the conditional mean attendance rate. Compared with a fully parametric generalized Poisson model [30], this approach adjusts standard errors using a moment-based dispersion parameter without requiring specification of a particular count distribution.

The model included five weekday indicators (Tuesday–Saturday, with Monday as the reference), attendance on the same weekday 14 calendar days earlier (lag-14 attendance), and same-day atmospheric pressure. The weekday indicators and lag-14 attendance represented the dominant institutional scheduling structure. The lag-14 term also captured persistence associated with the biweekly program rotation and habitual attendance and was treated as an adjustment term rather than interpreted substantively.

Days for which the date 14 days earlier was a non-operating day or fell in the preceding fiscal year were excluded. This resulted in exclusion of approximately 10% of operating days per fiscal year. The association between atmospheric pressure and attendance was summarized using incidence rate ratios (IRRs) with 95% confidence intervals (CIs). Statistical significance was assessed using two-sided α = 0.05, with p-values interpreted descriptively because the analyses were exploratory.

#### Robustness to adjustment and alternative specifications.

The robustness of the pressure association was evaluated primarily from the stability of its direction, magnitude, and precision across a series of pre-planned and exploratory stress tests. Changes in whether individual p-values crossed the 0.05 threshold were reported descriptively but were not used as a binary criterion for robustness.

First, the primary model was re-estimated using heteroskedasticity- and autocorrelation-consistent standard errors (HAC/Newey–West [31], bandwidth 4).

Second, co-varying meteorological factors were introduced in separate adjustment models: (a) temperature and relative humidity, (b) precipitation, and (c) atmospheric pressure 14 days earlier. These analyses assessed whether the same-day pressure estimate remained similar after accounting for meteorological conditions that could co-vary with pressure or with attendance.

Third, because mood and psychotic disorders show well-documented seasonal variation [32], seasonal robustness was assessed using three alternative representations of within-year seasonality: eleven calendar-month indicators, two pairs of annual Fourier sine–cosine harmonic terms, and a natural cubic spline with four degrees of freedom for the day index within the fiscal year. Using multiple seasonal representations reduced dependence on any single parameterization.

Fourth, exploratory sensitivity analyses replaced same-day pressure with lag-1, lag-2, and lag-3 pressure and with 3-day (lags 0–2) and 4-day (lags 0–3) mean pressure. These analyses were not used to redefine the primary candidate predictor.

Fifth, because the lag-14 attendance term is a strong proxy for the scheduling structure and recent attendance history, the primary model was re-estimated without this term, leaving weekday indicators as the structural adjustment. This sensitivity analysis examined whether inclusion of lag-14 attendance materially altered the pressure estimate.

Following residual inspection, an additional post hoc model including lag-1 attendance was fitted for FY2022 because that year showed greater residual autocorrelation than the other fiscal years. This analysis was exploratory and was not used to redefine the primary analysis.

For the meteorological and seasonal adjustment models, variance inflation factors (VIFs) were calculated from the same design matrices used for estimation, including the intercept, weekday indicators, lag-14 attendance, atmospheric pressure, and the relevant meteorological or seasonal adjustment terms. VIFs were used to assess whether coefficient instability could plausibly reflect problematic multicollinearity. In the natural cubic spline models, only the pressure VIF was interpreted because the individual spline basis functions are structurally linearly dependent with the intercept under this coding, making conventional term-wise VIFs for the basis functions non-informative.

#### Incremental predictive utility.

Incremental predictive utility was assessed by comparing models with and without atmospheric pressure using out-of-sample predictions. Because the candidate predictor was same-day daily mean atmospheric pressure, which is not fully observed until the day is complete, these analyses were intended to quantify out-of-sample predictive utility rather than prospective operational forecasting.

The primary predictive assessment used five-fold expanding-window time-series cross-validation separately within each fiscal year, preserving temporal ordering between training and held-out observations. A structural scheduling model containing weekday indicators and lag-14 attendance was compared with an otherwise identical model that additionally included same-day atmospheric pressure. Held-out predictions were pooled across folds and evaluated using cross-validated R^2^ and mean absolute error (MAE). For R^2^, each fold’s training-set mean served as the reference. The incremental predictive contribution of atmospheric pressure was expressed as ΔR^2^ between the full and scheduling models. This expanding-window analysis was the primary temporally ordered validation.

A secondary sensitivity analysis assessed whether pressure added predictive information beyond a baseline that already incorporated seasonality. Repeated five-fold cross-validation with ten repeats was used to compare models with and without atmospheric pressure after annual Fourier harmonic terms had been added to the structural baseline. Repeated K-fold rather than expanding-window cross-validation was used for this comparison because annual seasonal terms were unstable when estimated from the short initial training windows required by forward validation. Because the interleaved K-fold partitions did not preserve temporal ordering, this analysis was treated as a secondary non-temporal resampling comparison rather than as temporal validation of future observations. The seasonal terms were deterministic functions of calendar date, and each fold spanned the annual cycle.

As an additional sensitivity analysis, out-of-sample performance was also examined for models without the lag-14 attendance term, using weekday indicators as the structural baseline.

#### Model diagnostics and cross-year consistency.

Pearson-residual autocorrelation was examined at lags 1–20. Additional diagnostics included the Durbin–Watson statistic [33], Ljung–Box Q statistics [34] at lags 5 and 10, residuals-versus-fitted plots, normal Q–Q plots, and the Shapiro–Wilk test of residual normality.

The primary model was fitted independently in each fiscal year. The direction and magnitude of the atmospheric-pressure coefficient were compared across years, and between-year heterogeneity was evaluated using Cochran’s Q and the I^2^ statistic. These cross-year comparisons were used to characterize consistency of the primary estimates rather than to establish generalizability beyond the single study site.

### Software and reproducibility

Analyses were conducted using Python 3.12.3 with statsmodels 0.14.6 for quasi-Poisson generalized linear models, residual diagnostics, and HAC standard errors; scikit-learn 1.8.0 for cross-validation partitioning; SciPy 1.17.1 for the Shapiro–Wilk test; patsy 1.0.2 for seasonal design matrices; and NumPy 2.4.4 and pandas 3.0.2 for data handling. Figures were produced using matplotlib 3.10.8.

The anonymized analytic dataset and analysis code reproducing the reported results are publicly available in the Zenodo repository (https://doi.org/10.5281/zenodo.20518605).

### Use of generative AI

During the preparation of this work, the authors used a generative AI assistant for assistance with the statistical analysis code and to improve the English language of the manuscript. After using this tool, the authors reviewed and edited the content as needed and take full responsibility for the content of the published article.

## Results

### Baseline characteristics

The facility operated on 295, 294, 293, and 293 days in FY2022–FY2025, respectively. After excluding days for which a valid lag-14 attendance value was unavailable because the corresponding date was a non-operating day or fell in the preceding fiscal year, the analytic samples comprised 266, 264, 263, and 262 operating days.

Mean daily attendance was 17.6 (SD 4.64), 15.7 (4.30), 17.8 (4.30), and 14.7 (4.24) persons in FY2022–FY2025, respectively. Mean same-day atmospheric pressure was 1014.6 (SD 6.74), 1013.9 (6.28), 1012.9 (6.53), and 1014.4 (6.38) hPa. Participant characteristics for FY2025 and operating-day statistics for all four fiscal years are summarized in Table 1.

### Detectability: A positive pressure–attendance association across four fiscal years

After adjustment for weekday scheduling and same-weekday attendance 14 days earlier, same-day atmospheric pressure was positively associated with daily attendance in each of the four fiscal years (Table 2). The estimated pressure IRRs were 1.0037 (95% CI 1.0010–1.0065; *p* = 0.007) in FY2022, 1.0033 (1.0002–1.0064; *p* = 0.039) in FY2023, 1.0032 (1.0005–1.0059; *p* = 0.020) in FY2024, and 1.0069 (1.0040–1.0098; *p* < 0.001) in FY2025. Thus, the direction of association was consistent across years and met the conventional threshold for statistical significance in each primary model.

**Table 2:** Primary quasi-Poisson GLM: same-day atmospheric pressure and same-weekday attendance 14 days earlier (FY2022–FY2025).

FY	n	Pressure IRR [95% CI]	p	Lag-14 IRR [95% CI]	p	φ
2022	266	1.0037 [1.0010, 1.0065]	0.007	1.0243 [1.0183, 1.0303]	<0.001	0.370
2023	264	1.0033 [1.0002, 1.0064]	0.039	1.0075 [1.0004, 1.0146]	0.040	0.400
2024	263	1.0032 [1.0005, 1.0059]	0.020	1.0106 [1.0049, 1.0164]	<0.001	0.372
2025	262	1.0069 [1.0040, 1.0098]	<0.001	1.0208 [1.0134, 1.0282]	<0.001	0.317

*Note.* The expected change in daily attendance per +10 hPa increase in pressure, derived from the fitted IRRs, was approximately 0.7 (FY2022), 0.5 (FY2023), 0.6 (FY2024), and 1.0 (FY2025) persons.

The estimated magnitude was modest. A 10-hPa increase in pressure corresponded to an expected difference of approximately 0.5–1.0 persons per operating day, equivalent to roughly a 3%–7% difference in expected attendance. Across an approximate within-year 10th-to-90th-percentile pressure range of 17 hPa, this corresponded to about 0.9–1.8 persons, or approximately 5%–12% of expected attendance, below the observed day-to-day standard deviations of approximately 4–5 persons.

Lag-14 attendance was also positively associated with current attendance in each year, consistent with the biweekly program rotation. The quasi-Poisson dispersion parameter was below 1 in all four fiscal years (φ = 0.32–0.40), indicating systematic underdispersion in daily attendance counts.

### Robustness: Sensitivity to co-varying meteorological factors and seasonality

Although the pressure association was statistically detectable in all four primary models, the robustness of the estimated effect varied across fiscal years when judged from the stability of its direction, magnitude, and precision under adjustment (Tables 3 and 4).

**Table 3:** Same-day pressure IRR (95% CI; p-value) under seasonal adjustment.

FY	Primary	+ Month	+ Harmonic	+ Spline
2022	1.0037 [1.0010, 1.0065] (0.007)	0.9990 [0.9961, 1.0019] (0.501)	0.9997 [0.9968, 1.0026] (0.846)	0.9998 [0.9971, 1.0026] (0.912)
2023	1.0033 [1.0002, 1.0064] (0.039)	1.0049 [1.0012, 1.0087] (0.009)	1.0057 [1.0022, 1.0093] (0.002)	1.0053 [1.0018, 1.0089] (0.003)
2024	1.0032 [1.0005, 1.0059] (0.020)	1.0026 [0.9995, 1.0057] (0.095)	1.0034 [1.0003, 1.0065] (0.033)	1.0035 [1.0005, 1.0064] (0.021)
2025	1.0069 [1.0040, 1.0098] (<0.001)	1.0065 [1.0031, 1.0099] (<0.001)	1.0056 [1.0024, 1.0088] (<0.001)	1.0064 [1.0032, 1.0096] (<0.001)

**Table 4:** Same-day pressure IRR (95% CI; p-value) under other adjustments and robust standard errors.

FY	+ Precipitation	+ Temp & humidity	+ Lag-14 pressure	HAC SE
2022	1.0032 [1.0004, 1.0060] (0.024)	1.0003 [0.9976, 1.0031] (0.814)	1.0028 [1.0001, 1.0055] (0.045)	1.0037 [1.0008, 1.0067] (0.012)
2023	1.0015 [0.9984, 1.0046] (0.357)	1.0056 [1.0019, 1.0092] (0.003)	1.0049 [1.0017, 1.0081] (0.003)	1.0033 [1.0004, 1.0061] (0.024)
2024	1.0025 [0.9998, 1.0052] (0.070)	1.0047 [1.0018, 1.0076] (0.001)	1.0032 [1.0005, 1.0059] (0.019)	1.0032 [1.0000, 1.0064] (0.050)
2025	1.0065 [1.0036, 1.0094] (<0.001)	1.0063 [1.0032, 1.0095] (<0.001)	1.0067 [1.0038, 1.0096] (<0.001)	1.0069 [1.0041, 1.0097] (<0.001)

*Note.* Precipitation was entered as same-day rainfall (mm). Its coefficient in the “+ Precipitation” model (IRR per mm [95% CI]) was 0.9988 [0.9972, 1.0004], *p* = 0.155 (FY2022); 0.9973 [0.9961, 0.9986], *p* < 0.001 (FY2023); 0.9979 [0.9965, 0.9993], *p* = 0.003 (FY2024); and 0.9987 [0.9973, 1.0000], *p* = 0.058 (FY2025). An IRR below 1 indicates lower attendance on rainier days.

Seasonal adjustment had the largest effect on magnitude in FY2022, reducing the pressure estimate from the primary IRR of 1.0037 to approximately the null under calendar-month indicators (IRR 0.9990, *p* = 0.501), Fourier harmonics (IRR 0.9997, *p* = 0.846), and a natural cubic spline of within-year time (IRR 0.9998, *p* = 0.912). In FY2024, the point estimate was comparatively stable across seasonal representations (IRR 1.0026–1.0035 versus 1.0032 in the primary model), but precision was specification-sensitive: the month-adjusted result had *p* = 0.095, whereas the harmonic and spline results had *p* = 0.033 and *p* = 0.021, respectively. In FY2023 and FY2025, the seasonally adjusted point estimates remained positive and of similar or somewhat larger magnitude than the corresponding primary estimates.

Adjustment for precipitation reduced the pressure estimate in FY2023 from IRR 1.0033 to 1.0015 (*p* = 0.357) and in FY2024 from 1.0032 to 1.0025 (*p* = 0.070). Precipitation itself was negatively associated with attendance and reached conventional statistical significance in FY2023 and FY2024. Adjustment for temperature and relative humidity reduced the FY2022 pressure estimate from 1.0037 to near the null (IRR 1.0003, *p* = 0.814), whereas the estimates in the other years remained positive and of similar or larger magnitude than their primary estimates.

Adjustment for atmospheric pressure 14 days earlier produced little change in the same-day pressure estimates. Likewise, using HAC/Newey–West standard errors left the point estimates unchanged while altering their precision; for FY2024, the corresponding p-value was 0.050.

Taken together, effect-estimate stability differed across years. FY2022 showed marked attenuation of the pressure estimate toward the null under seasonal and temperature–humidity adjustment; FY2023 showed moderate attenuation after precipitation adjustment; FY2024 retained a broadly similar point estimate across most specifications but with specification-sensitive precision; and FY2025 showed the greatest stability in both magnitude and precision across the adjustment sets. Conventional statistical significance was retained across all adjustment specifications only in FY2025, but this threshold pattern was not used as a binary criterion for robustness. The observed sensitivity does not by itself identify its cause: co-variation among meteorological factors, measurement error, seasonal structure, and sampling variability could all contribute.

Variance-inflation diagnostics provided no evidence of problematic multicollinearity. Across all meteorological and seasonal adjustment models, the VIF for atmospheric pressure ranged from 1.05 to 1.65. VIFs for the other meteorological or seasonal adjustment terms were ≤2.6 where conventional term-wise VIFs were defined. In the natural cubic spline models, the pressure VIF was 1.24–1.41; individual spline-basis VIFs were not interpreted for the structural reason described in the Methods. Thus, the marked FY2022 attenuation after seasonal adjustment was not accompanied by substantial variance inflation in the pressure coefficient.

Post hoc examination showed that the correlation between same-day pressure and precipitation was weakest in FY2025 (r = −0.09, compared with −0.17 to −0.28 in the other fiscal years), consistent with less overlap between these predictors in that year. In FY2024, the pressure point estimate changed relatively little across several adjustment models while its confidence interval widened toward the null; the result is therefore better characterized as attenuated and uncertain than as indicating a qualitative presence-or-absence change.

Removing lag-14 attendance from the model did not materially reduce the same-day pressure estimates. The corresponding IRRs without lag-14 were 1.0070, 1.0028, 1.0038, and 1.0085 in FY2022–FY2025, compared with 1.0037, 1.0033, 1.0032, and 1.0069 in the primary models (S3 Table). Thus, the observed sensitivity of the pressure estimate under other adjustments was not explained by attenuation through inclusion of lag-14 attendance.

The alternative-pressure-exposure analyses are summarized in S2 Table. Associations generally attenuated at longer individual lags, and none of the alternative lagged or multi-day pressure measures produced a consistently stronger pattern across fiscal years.

### Predictive utility: Negligible and unstable incremental out-of-sample gain

The statistically detectable pressure association did not translate into a stable improvement in out-of-sample prediction (Table 5).

**Table 5:** Incremental predictive utility of atmospheric pressure (ΔR^2^ from adding same-day pressure).

FY	Forward-CV R^2^ (sched.)	ΔR^2^ (forward)	K-fold ΔR^2^ (sched. base)	K-fold ΔR^2^ (sched. + season)
2022	0.667	+0.003	+0.004	−0.002
2023	0.582	+0.003	+0.001	+0.007
2024	0.553	−0.018	+0.003	+0.003
2025	0.693	+0.030	+0.025	+0.017
Mean	—	+0.005	+0.008	+0.006

*Note.* Forward CV preserved temporal ordering and was the primary predictive assessment. Repeated K-fold estimates were secondary non-temporal resampling sensitivity analyses and were not interpreted as temporal forecasting validation.

In five-fold expanding-window time-series cross-validation, the scheduling model containing weekday indicators and lag-14 attendance achieved cross-validated R^2^ values of 0.667, 0.582, 0.553, and 0.693 in FY2022–FY2025, respectively. Adding same-day atmospheric pressure changed R^2^ by +0.003, + 0.003, −0.018, and +0.030, respectively, giving a mean ΔR^2^ of +0.005 across the four years.

A secondary, non-temporal repeated K-fold sensitivity analysis yielded similarly small increments. Relative to the scheduling baseline, mean ΔR^2^ values were +0.004, + 0.001, + 0.003, and +0.025 for FY2022–FY2025, respectively (overall mean +0.008). When annual seasonality was also included in the baseline, the corresponding increments were −0.002, + 0.007, + 0.003, and +0.017 (overall mean +0.006). These K-fold results were used as resampling-based sensitivity comparisons and were not interpreted as temporal forecasting validation.

In this secondary non-temporal analysis, across the 50 repeated folds for the scheduling-baseline comparison, the 95% empirical interval for ΔR^2^ included zero in every fiscal year: FY2022, + 0.004 [−0.045, + 0.022]; FY2023, + 0.001 [−0.038, + 0.018]; FY2024, + 0.003 [−0.043, + 0.027]; and FY2025, + 0.025 [−0.029, + 0.064]. Fold-to-fold standard deviations ranged from 0.016 to 0.024 and were comparable to or larger than the mean incremental gain in each year.

In absolute-error terms, adding atmospheric pressure changed cross-validated MAE by only −0.06 to +0.04 persons per operating day, corresponding to approximately −3% to +2% of the scheduling-model error. Thus, although some fiscal years showed small positive increments, these gains were neither large nor stable across years; the secondary non-temporal resampling analyses did not alter that interpretation.

The same qualitative conclusion was obtained when lag-14 attendance was omitted from the structural baseline. The weekday-only forward-cross-validated ΔR^2^ values were +0.033, −0.006, −0.015, and +0.049 in FY2022–FY2025, respectively (S3 Table). Pressure therefore provided no consistently useful incremental predictive signal regardless of whether recent attendance history was included in the baseline.

### Model diagnostics and cross-year consistency

Residual diagnostics indicated that the primary model captured temporal structure more completely in FY2023–FY2025 than in FY2022 (Fig 2). Durbin–Watson statistics were 1.67, 1.92, 1.82, and 1.87 for FY2022–FY2025, respectively. The Ljung–Box Q statistic at lag 10 was non-significant in FY2023 (*p* = 0.737) and FY2024 (*p* = 0.271). FY2025 showed evidence of residual autocorrelation at shorter lags, including a significant Ljung–Box statistic at lag 5 (*p* = 0.005). FY2022 showed the greatest residual temporal dependence, with Ljung–Box statistics significant at lags 5 and 10 (both *p* < 0.001). Residual distributions were approximately normal except in FY2024, for which the Shapiro–Wilk test gave *p* = 0.004 (S1 Fig).

**Fig 2:**
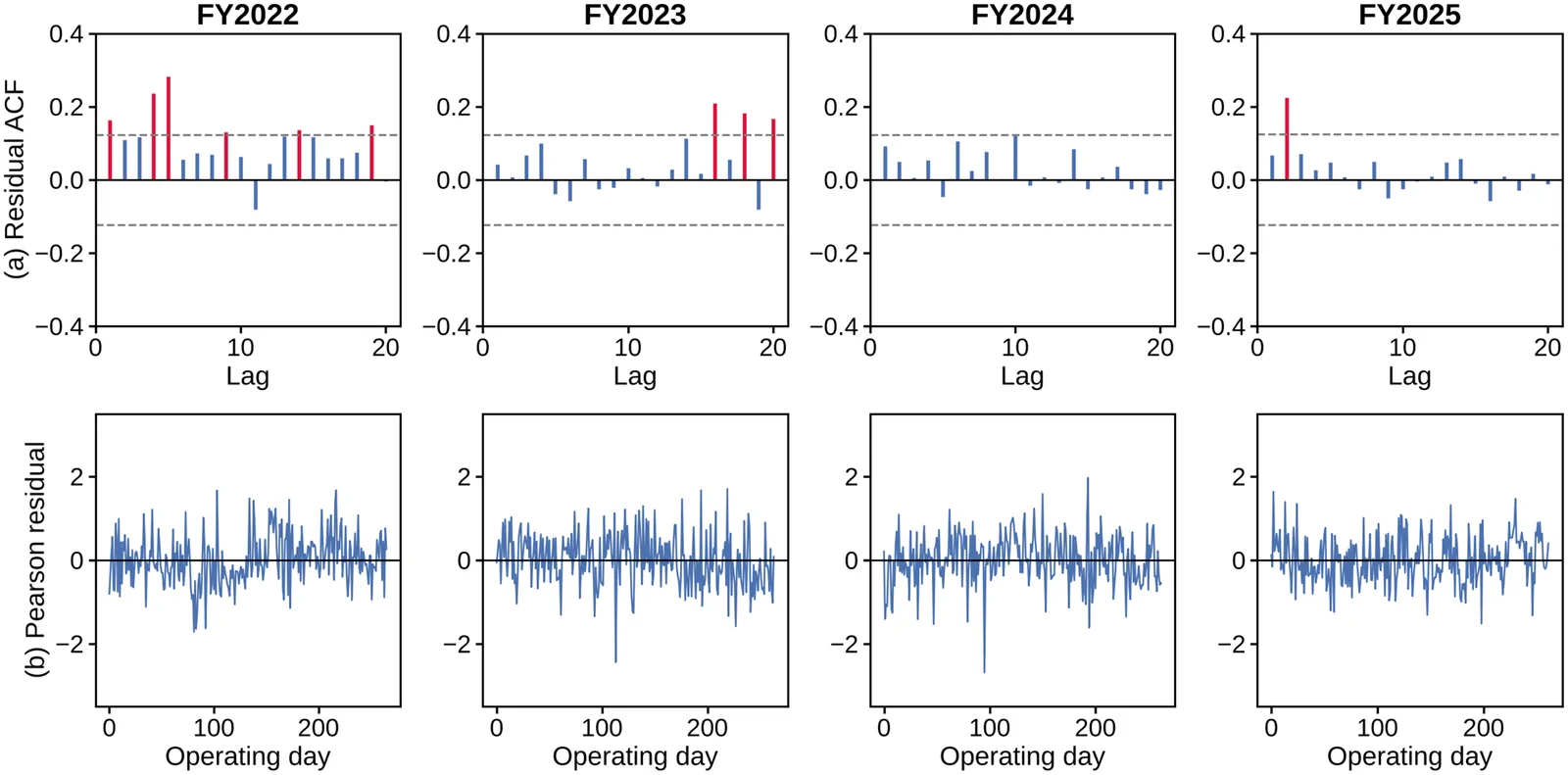
Residual autocorrelation diagnostics for the primary quasi-Poisson GLM across four fiscal years. Rows: (a) Pearson-residual autocorrelation (lags 1–20; dashed band ±2/√n, significant lags in red); (b) Pearson residuals over operating days. Residuals-versus-fitted and normal Q–Q plots appear in S1 Fig.

Because FY2022 showed greater residual autocorrelation, a post hoc model adding lag-1 attendance was examined. In this model, the FY2022 pressure estimate decreased to IRR 1.0027 (*p* = 0.087). This exploratory result was not used to redefine the primary model but further indicated sensitivity of the FY2022 estimate to additional temporal adjustment.

Despite these differences in robustness, the primary pressure coefficients did not show statistically significant between-year heterogeneity. Cochran’s Q was 4.40 (df = 3, *p* = 0.222), with I^2^ = 31.7% and a pooled fixed-effect IRR of 1.0042. Thus, the primary estimates were statistically compatible across years, even though their sensitivity to meteorological and seasonal adjustment differed substantially.

## Discussion

### Principal findings

Using four consecutive fiscal years from a single psychiatric day-care, we evaluated one pre-specified candidate predictor through three distinct questions: whether its association with attendance was statistically detectable, whether its estimated direction, magnitude, and precision were robust to reasonable adjustment and alternative specifications, and whether it provided incremental out-of-sample predictive utility beyond a strong structural baseline. Same-day atmospheric pressure was positively associated with attendance in the primary model in all four fiscal years. However, effect-estimate robustness varied across years: FY2022 showed marked attenuation toward the null under seasonal and temperature–humidity adjustment, FY2023 showed moderate attenuation after precipitation adjustment, FY2024 retained broadly similar point estimates with specification-sensitive precision, and FY2025 showed the greatest stability across adjustment sets. Nor did detectability translate into substantial incremental predictive utility. In the primary expanding-window temporal evaluation, adding atmospheric pressure produced only small, unstable changes in performance, whereas secondary non-temporal K-fold resampling analyses yielded similarly small increments.

The central finding is therefore not a particular biometeorological effect of atmospheric pressure. Rather, this worked example shows that the same candidate predictor can appear consistently detectable while its estimated effect is sensitive in magnitude or precision to reasonable adjustment and its incremental out-of-sample predictive utility remains limited. These properties answer different questions and should not be treated as interchangeable evidence.

### Detectability, robustness, and predictive utility answer different questions

Statistical detectability, as used here, indicates that an association meets a prespecified inferential threshold under a specified model; it does not establish that the coefficient represents an independent signal or that the variable improves prediction. The present results illustrate both distinctions within the same dataset. The pressure coefficient met the conventional significance threshold in each of the four primary annual models, yet its magnitude or precision changed when related meteorological and seasonal information was introduced, and its addition to the structural prediction model produced no stable improvement in the primary expanding-window held-out evaluation. This is consistent with broader cautions against treating statistical significance as a sufficient basis for substantive interpretation [35].

This distinction complements the broader separation between explanation and prediction [1]. An explanatory analysis asks how an outcome varies with a candidate factor conditional on a specified set of variables, whereas a predictive analysis asks whether that factor improves performance on observations not used for fitting. Robustness adds a further question: whether the direction, magnitude, and precision of the estimated association remain reasonably stable after plausible co-varying factors and alternative reasonable specifications are considered. Changes in whether a p-value crosses 0.05 are therefore descriptive rather than a binary definition of robustness. A predictor can be statistically detectable without satisfying either of the latter criteria.

The present example also illustrates why the baseline model matters when judging predictive utility. Attendance in this day-care was highly structured by assigned weekdays, the biweekly program rotation, and recent attendance history. The scheduling baseline consequently explained 55%–69% of out-of-sample variance before atmospheric pressure was added. This limited the amount of residual predictable variation available to any environmental variable. The appropriate interpretation is therefore not that atmospheric pressure contained exactly zero predictive information, but that its incremental contribution beyond the information already available from scheduling and recent attendance was small and unstable in out-of-sample evaluation.

More generally, a statistically significant candidate predictor should not be promoted directly from association to substantive interpretation or practical use. Where feasible, its interpretation can be strengthened by asking sequentially whether the signal is detectable, whether its estimated direction, magnitude, and precision remain reasonably stable under appropriate stress tests, and whether it adds out-of-sample information beyond an appropriate baseline. The present single-site study illustrates the usefulness of this sequence; it does not establish that the same three-stage pattern will occur for other predictors or in other clinical settings.

### Interpreting the atmospheric-pressure example

Because atmospheric pressure served primarily as a worked example, its substantive interpretation should remain cautious. Several observations argue against treating the primary coefficient as evidence of a specific physiological pressure effect. The estimate was sensitive to different adjustments in different years. Precipitation attenuated the pressure estimate particularly in FY2023 and FY2024, temperature and humidity substantially attenuated it in FY2022, and seasonal adjustment moved the FY2022 estimate to approximately the null while reducing precision in some FY2024 specifications. These patterns are compatible with pressure capturing part of a broader meteorological or seasonal pattern rather than a distinct pressure-specific effect.

However, attenuation after adjustment does not identify a unique explanation. Variance-inflation diagnostics argued against problematic multicollinearity as a major numerical artifact: the VIF for atmospheric pressure ranged from 1.05 to 1.65 across all meteorological and seasonal adjustment models, and other adjustment terms had VIFs ≤ 2.6 where conventional term-wise VIFs were defined. In the spline models, the pressure VIF likewise remained low (1.24–1.41), while individual basis-function VIFs were not interpreted because of their structural dependence with the intercept. These diagnostics make substantial variance inflation an unlikely explanation for the FY2022 attenuation after seasonal adjustment. Nevertheless, low VIFs do not eliminate shared meteorological or seasonal structure. Genuine confounding by co-varying conditions, measurement error, and year-to-year sampling variability therefore remain plausible explanations for changes in the pressure estimate. We therefore do not interpret the adjusted models as establishing that precipitation, temperature, humidity, or seasonality is the “true” causal factor replacing atmospheric pressure.

One interpretation consistent with, but not established by, the data is that higher atmospheric pressure partly indexes settled weather conditions that co-occur with other environmental characteristics relevant to attendance. Such an interpretation would be compatible with the inconsistent pressure–psychiatry literature and the absence of an established mechanism [11,12]. It also avoids attributing the observed association specifically to a physiological response to barometric pressure. Experimental work on transient barometric changes has focused on sensory or pain responses rather than psychiatric service use [36,37]. The broader literature itself does not provide a simple alternative causal explanation: for example, associations between precipitation and psychiatric outcomes have also been inconsistent [38].

FY2025 requires particular caution because it showed the greatest stability of the pressure estimate across adjustment models and was also the only fiscal year in which conventional statistical significance was retained under every adjustment examined. The latter threshold-based pattern is not our criterion for robustness, but it could be overinterpreted if viewed without considering multiplicity. The analyses were exploratory, involved four fiscal years and multiple related specifications, and were conducted without formal multiplicity correction. As an illustrative conservative calculation under a global null, if each individual test is calibrated at alpha = 0.05, the probability that a given year would remain statistically significant under every adjustment cannot exceed 0.05, regardless of the dependence among the adjustment models. If the four fiscal years are additionally treated as independent for illustration, setting this per-year probability at its maximum of 0.05 gives a probability of exactly one surviving year of 4(0.05)(0.95)^3^ = 0.171, and a probability of at least one surviving year of 1 − (0.95)^4^ = 0.185. These values are illustrative benchmarks rather than formal probabilities: the adjustment models were fitted to the same observations and shared most covariates, and the fiscal years cannot be assumed to be strictly exchangeable or independent. A precise null probability would require a resampling or simulation procedure reproducing the full analysis pipeline. This calculation pertains only to the binary significance pattern, not to robustness of the effect estimate itself. It nevertheless shows that a single year retaining significance across all adjustments is not sufficiently improbable under chance alone to constitute strong evidence of a year-specific effect. We therefore continue to regard the FY2025 finding as weak and exploratory.

Other explanations also remain possible. FY2025 showed weaker correlation between pressure and precipitation than the other years, so adjustment removed less overlapping variation. It also had the largest primary pressure estimate and the largest, although still small, increment in the primary expanding-window predictive assessment. A small genuine year-specific effect therefore cannot be excluded. Nevertheless, the annual confidence intervals overlapped widely, between-year heterogeneity of the primary estimates was not statistically significant, and the predictive gain did not recur consistently. We therefore regard FY2025 as unresolved rather than as evidence of a reproducible pressure effect.

### Setting dependence and generalizability

The limited predictive contribution of weather should be interpreted in relation to the unusually strong structural constraints on attendance in this setting. Patients attended on pre-assigned weekdays within a biweekly program rotation, and recent same-weekday attendance was highly informative. In addition, a relatively high proportion of patients traveled by private car, with no shuttle service. These features characterize a suburban Japanese psychiatric day-care and may reduce the influence of daily weather on whether a registered patient attends.

The findings should therefore not be generalized to psychiatric service use as a whole. Weather could have greater predictive relevance in settings where attendance is more discretionary, scheduling is flexible, or patients depend more heavily on walking or public transportation. The contrast may be still greater for emergency and inpatient services, where presentation is driven more directly by acute changes in symptoms and where most previous weather–psychiatry studies have been conducted [2–15]. The present results characterize the structure-dominated end of this continuum rather than establishing that weather lacks relevance for psychiatric service utilization generally.

This distinction also limits the methodological generalization. The study demonstrates one concrete instance in which detectability, robustness, and predictive utility diverged; a single facility cannot validate this three-part evaluation as universally transferable. Multi-site applications using different candidate predictors, service structures, and populations would be needed to determine how often similar divergences occur.

### Applications of the framework

The three-stage sequence is not specific to meteorological exposures. It applies whenever a candidate variable is proposed as a meaningful or actionable correlate of a routinely recorded outcome. In health-service demand forecasting, predictors such as weather, air quality, public holidays, local events, or internet search activity are regularly reported to be associated with attendance, admissions, or emergency presentations; the sequence asks in turn whether such an association is detectable once the calendar information and recent history already available to the service are accounted for, whether the estimate is stable under reasonable adjustment, and whether acquiring and maintaining an external data feed would measurably improve forecasts. In clinical prediction modeling, the same questions arise when a new marker is proposed for addition to an established model, where incremental value beyond existing predictors is the accepted criterion [27]. The sequence is equally relevant to signals derived from wearable devices and smartphone use in mental health research, where statistically significant associations with routinely collected outcomes are often followed quickly by operational claims.

Two features make the sequence straightforward to adopt. First, it requires no specialized methodology: a regression model appropriate to the outcome, adjustment for plausible co-varying factors together with collinearity diagnostics, and out-of-sample cross-validation are standard tools, and these analyses cost little beyond the model that would be fitted in any case. Second, what the third stage can show depends on how the baseline is specified. A baseline containing only the candidate predictor will almost always suggest an apparent gain, whereas a baseline containing the information the setting already holds asks the question that matters operationally. Reporting that baseline explicitly allows readers to judge what any incremental gain means, and makes clear when a small increment reflects a weak predictor rather than an already strong baseline.

### Limitations

Several limitations should be considered. First, this was a single-facility study, and the participant population and service organization may not represent other psychiatric day-care programs in Japan or elsewhere. The FY2025 registered cohort was modest in size and included heterogeneous psychiatric diagnoses. Because the outcome was an aggregate facility-level daily count, the analysis cannot determine whether particular diagnostic, demographic, or transport-defined subgroups are individually sensitive to weather, a general limitation of ecological inference [39]. A clinically meaningful association confined to a small weather-sensitive subgroup could therefore be attenuated or entirely masked in the aggregate outcome.

Second, the analyses were exploratory and were not pre-registered. Although atmospheric pressure and the covariate set were fixed during planning and were not selected after inspection of the results, repeated analyses were conducted across four fiscal years, several adjustment sets, seasonal representations, and alternative lag structures without formal multiplicity correction. P-values should therefore be interpreted descriptively, and isolated findings close to conventional significance thresholds warrant particular caution.

Third, the lag-14 attendance covariate jointly captures several features, including the biweekly program rotation, serial dependence, and habitual attendance, and these components cannot be separated. Its inclusion may aggressively absorb temporally structured variation. However, removing lag-14 did not materially reduce the estimated pressure coefficient and did not yield a stable predictive contribution from pressure, suggesting that the principal conclusions did not depend on this adjustment.

Fourth, residual autocorrelation was greater in FY2022 than in the other fiscal years. This period overlapped with the later phase of the COVID-19 pandemic in Japan, when infection-control measures could have produced irregular attendance disruptions unrelated to weather. In a post hoc analysis adding lag-1 attendance, the FY2022 pressure estimate decreased to IRR 1.0027 (*p* = 0.087). This further indicates sensitivity of the FY2022 estimate to temporal adjustment but should be regarded as exploratory.

Fifth, same-day pressure was the pre-specified candidate predictor. Delayed or cumulative meteorological effects are possible, but exploratory analyses of lagged and multi-day pressure measures did not reveal a consistently stronger association or a stable predictive gain. The absence of incremental predictive utility was therefore not confined to the lag-0 specification examined in the primary analysis. In addition, same-day daily mean pressure is not fully observed until the day is complete. Accordingly, the predictive analyses quantify out-of-sample predictive utility under contemporaneous exposure information and should not be interpreted as prospective operational forecasts. A deployable forecasting application would require meteorological predictors available at the time the prediction is made, such as prior observations or weather forecasts. Other potentially relevant meteorological dimensions, such as sunshine duration and wind conditions, were not evaluated.

Sixth, meteorological exposure was assigned from representative observation stations rather than measured at the facility. Atmospheric pressure and humidity were obtained from the Chiba Local Meteorological Observatory approximately 20 km away, whereas temperature and precipitation were obtained from the nearer Kisarazu station. Atmospheric pressure generally varies relatively little over such a distance, but using different stations for pressure and precipitation could introduce exposure mismatch and attenuate their observed relationship.

Finally, the ecological observational design does not support causal inference. Attenuation or loss of precision after adjustment should therefore be interpreted as evidence that the pressure estimate is specification-sensitive, not as proof of which meteorological factor, if any, causally influences attendance.

## Conclusion

In this single, highly schedule-driven psychiatric day-care, same-day atmospheric pressure was statistically detectable as a correlate of daily attendance across four fiscal years, but effect-estimate stability varied across years, with marked attenuation in FY2022, moderate attenuation after precipitation adjustment in FY2023, and specification-sensitive precision in FY2024, whereas FY2025 was comparatively stable; atmospheric pressure also provided little stable incremental out-of-sample predictive value beyond scheduling variables and recent attendance history.

The substantive conclusion is therefore limited to this setting: the weather signal examined added little useful information for out-of-sample prediction of aggregate attendance once the dominant structural predictors were taken into account. These findings should not be generalized to psychiatric services in which attendance is more discretionary or symptom-driven.

More broadly, this worked example illustrates a methodological distinction. Statistical detectability, robustness to reasonable adjustment, and predictive utility are separate properties of a candidate predictor and should be evaluated as such. A statistically significant association should not be interpreted as independently meaningful or practically actionable without considering whether its direction, magnitude, and precision remain reasonably stable under appropriate stress tests and whether it improves prediction beyond an adequate baseline.

## Supporting information

S1 Fig Residuals-versus-fitted and normal Q–Q plots for the primary quasi-Poisson GLM across four fiscal years. Rows: (a) residuals versus fitted values (linear trend in red); (b) normal Q–Q plot.
(TIF)

S2 Fig Pressure incidence rate ratios (per hPa) with 95% confidence intervals across robustness-adjustment models by fiscal year. Models: primary; with precipitation; with temperature and humidity; with seasonality (two annual Fourier harmonic pairs, as in Table 3); with lag-14 pressure; and the primary model under HAC/Newey–West robust standard errors. The dashed line marks no association (IRR = 1), and filled markers denote intervals excluding 1.
(TIF)

S1 Table Full regression results for the primary quasi-Poisson GLM—all coefficients, standard errors, p-values, 95% confidence intervals, and goodness-of-fit statistics—for each fiscal year. This is provided as a separate file.
(DOCX)

S2 Table Sensitivity of the same-day pressure association to alternative pressure exposures. Incidence rate ratios (p-value) for lag-1, lag-2, lag-3, and 3-day (lags 0–2) and 4-day (lags 0–3) mean pressure, alongside same-day (lag-0) pressure, by fiscal year, with the mean forward cross-validated ΔR^2^. The association generally attenuated at longer individual lags, and no exposure, including the multi-day means, yielded a stable predictive gain. This is provided as a separate file.
(DOCX)

S3 Table Sensitivity to the lag-14 attendance covariate. Same-day pressure incidence rate ratios (with 95% confidence intervals) estimated with and without the lag-14 term, and forward cross-validated R^2^ without the lag-14 term, by fiscal year. The pressure estimate was essentially unchanged and no stable predictive gain emerged. This is provided as a separate file.
(DOCX)
