# Supplementary material for: Evaluating a candidate predictor beyond statistical significance: Atmospheric pressure and psychiatric day-care attendance as a worked example
Source: PLoS One. 2026 Nov 6;21(11):e0352385. doi: 10.1371/journal.pone.0352385 (PMC13742932; doi:10.1371/journal.pone.0352385)
Supplement: Supplementary file 1 [file pone.0352385.s003.docx]

**S1 Table. Full regression results for the primary quasi-Poisson GLM**

Quasi-Poisson generalised linear model (log link; dispersion estimated by Pearson X2). Coefficients are on the log scale (beta); IRR = exp(beta). CI = 95% confidence interval. Goodness of fit: n analytic days, dispersion phi, residual and null deviance, and McFadden-type pseudo-R2 = 1 - residual/null deviance. One sub-table per fiscal year.

**FY2022**

| **Term** | **beta** | **SE** | **z** | **p** | **95% CI (beta)** | **IRR** | **95% CI (IRR)** |
| --- | --- | --- | --- | --- | --- | --- | --- |
| Intercept | -1.28579 | 1.38853 | -0.93 | 0.354 | [-4.00725, 1.43568] | 0.2764 | [0.0182, 4.2025] |
| Tuesday | -0.29155 | 0.03973 | -7.34 | <0.001 | [-0.36942, -0.21368] | 0.7471 | [0.6911, 0.8076] |
| Wednesday | -0.11200 | 0.03279 | -3.42 | <0.001 | [-0.17626, -0.04774] | 0.8940 | [0.8384, 0.9534] |
| Thursday | -0.09987 | 0.03334 | -3.00 | 0.003 | [-0.16522, -0.03452] | 0.9050 | [0.8477, 0.9661] |
| Friday | 0.09527 | 0.03179 | 3.00 | 0.003 | [0.03295, 0.15758] | 1.1000 | [1.0335, 1.1707] |
| Saturday | -0.01350 | 0.03040 | -0.44 | 0.657 | [-0.07308, 0.04609] | 0.9866 | [0.9295, 1.0472] |
| Attendance 14 d earlier (lag-14) | 0.02403 | 0.00299 | 8.05 | <0.001 | [0.01818, 0.02988] | 1.0243 | [1.0183, 1.0303] |
| Same-day pressure (per hPa) | 0.00372 | 0.00138 | 2.69 | 0.007 | [0.00101, 0.00643] | 1.0037 | [1.0010, 1.0065] |

Goodness of fit: n = 266; dispersion phi = 0.370; residual deviance = 95.63; null deviance = 320.01; pseudo-R2 = 0.701.

**FY2023**

| **Term** | **beta** | **SE** | **z** | **p** | **95% CI (beta)** | **IRR** | **95% CI (IRR)** |
| --- | --- | --- | --- | --- | --- | --- | --- |
| Intercept | -0.59279 | 1.61413 | -0.37 | 0.713 | [-3.75644, 2.57085] | 0.5528 | [0.0234, 13.0769] |
| Tuesday | -0.32336 | 0.04108 | -7.87 | <0.001 | [-0.40387, -0.24285] | 0.7237 | [0.6677, 0.7844] |
| Wednesday | -0.33174 | 0.04218 | -7.87 | <0.001 | [-0.41440, -0.24907] | 0.7177 | [0.6607, 0.7795] |
| Thursday | -0.17141 | 0.03703 | -4.63 | <0.001 | [-0.24400, -0.09882] | 0.8425 | [0.7835, 0.9059] |
| Friday | 0.18069 | 0.03511 | 5.15 | <0.001 | [0.11187, 0.24951] | 1.1980 | [1.1184, 1.2834] |
| Saturday | 0.05836 | 0.03314 | 1.76 | 0.078 | [-0.00660, 0.12333] | 1.0601 | [0.9934, 1.1313] |
| Attendance 14 d earlier (lag-14) | 0.00744 | 0.00362 | 2.06 | 0.040 | [0.00035, 0.01454] | 1.0075 | [1.0004, 1.0146] |
| Same-day pressure (per hPa) | 0.00327 | 0.00158 | 2.07 | 0.039 | [0.00017, 0.00636] | 1.0033 | [1.0002, 1.0064] |

Goodness of fit: n = 264; dispersion phi = 0.400; residual deviance = 104.39; null deviance = 304.20; pseudo-R2 = 0.657.

**FY2024**

| **Term** | **beta** | **SE** | **z** | **p** | **95% CI (beta)** | **IRR** | **95% CI (IRR)** |
| --- | --- | --- | --- | --- | --- | --- | --- |
| Intercept | -0.49183 | 1.37811 | -0.36 | 0.721 | [-3.19289, 2.20922] | 0.6115 | [0.0411, 9.1086] |
| Tuesday | -0.24457 | 0.03765 | -6.50 | <0.001 | [-0.31837, -0.17077] | 0.7830 | [0.7273, 0.8430] |
| Wednesday | -0.14026 | 0.03497 | -4.01 | <0.001 | [-0.20880, -0.07172] | 0.8691 | [0.8116, 0.9308] |
| Thursday | -0.09585 | 0.03428 | -2.80 | 0.005 | [-0.16304, -0.02865] | 0.9086 | [0.8496, 0.9718] |
| Friday | 0.19746 | 0.03685 | 5.36 | <0.001 | [0.12523, 0.26970] | 1.2183 | [1.1334, 1.3096] |
| Saturday | 0.02109 | 0.03332 | 0.63 | 0.527 | [-0.04422, 0.08639] | 1.0213 | [0.9567, 1.0902] |
| Attendance 14 d earlier (lag-14) | 0.01056 | 0.00292 | 3.62 | <0.001 | [0.00484, 0.01628] | 1.0106 | [1.0049, 1.0164] |
| Same-day pressure (per hPa) | 0.00317 | 0.00137 | 2.32 | 0.020 | [0.00050, 0.00585] | 1.0032 | [1.0005, 1.0059] |

Goodness of fit: n = 263; dispersion phi = 0.372; residual deviance = 98.01; null deviance = 263.82; pseudo-R2 = 0.628.

**FY2025**

| **Term** | **beta** | **SE** | **z** | **p** | **95% CI (beta)** | **IRR** | **95% CI (IRR)** |
| --- | --- | --- | --- | --- | --- | --- | --- |
| Intercept | -4.61210 | 1.48153 | -3.11 | 0.002 | [-7.51585, -1.70835] | 0.0099 | [0.0005, 0.1812] |
| Tuesday | -0.24968 | 0.03930 | -6.35 | <0.001 | [-0.32671, -0.17265] | 0.7790 | [0.7213, 0.8414] |
| Wednesday | -0.06084 | 0.03472 | -1.75 | 0.080 | [-0.12889, 0.00722] | 0.9410 | [0.8791, 1.0072] |
| Thursday | -0.17612 | 0.03652 | -4.82 | <0.001 | [-0.24770, -0.10455] | 0.8385 | [0.7806, 0.9007] |
| Friday | 0.21528 | 0.04030 | 5.34 | <0.001 | [0.13630, 0.29426] | 1.2402 | [1.1460, 1.3421] |
| Saturday | 0.09717 | 0.03432 | 2.83 | 0.005 | [0.02989, 0.16444] | 1.1020 | [1.0303, 1.1787] |
| Attendance 14 d earlier (lag-14) | 0.02054 | 0.00370 | 5.56 | <0.001 | [0.01330, 0.02779] | 1.0208 | [1.0134, 1.0282] |
| Same-day pressure (per hPa) | 0.00690 | 0.00147 | 4.69 | <0.001 | [0.00402, 0.00978] | 1.0069 | [1.0040, 1.0098] |

Goodness of fit: n = 262; dispersion phi = 0.317; residual deviance = 80.74; null deviance = 311.99; pseudo-R2 = 0.741.
