# Supplementary material for: Evaluating a candidate predictor beyond statistical significance: Atmospheric pressure and psychiatric day-care attendance as a worked example
Source: PLoS One. 2026 Nov 6;21(11):e0352385. doi: 10.1371/journal.pone.0352385 (PMC13742932; doi:10.1371/journal.pone.0352385)
Supplement: Supplementary file 2 [file pone.0352385.s004.docx]

**S2 Table. Sensitivity of the same-day pressure association to alternative pressure exposures.**

*Incidence rate ratio (p-value) for same-day pressure replaced by each exposure, by fiscal year, with the mean forward expanding-window cross-validated ΔR² (vs the scheduling baseline). Same model as the primary GLM (weekday indicators, lag-14 attendance).*

| **Pressure exposure** | **FY2022 IRR (p)** | **FY2023 IRR (p)** | **FY2024 IRR (p)** | **FY2025 IRR (p)** | **Mean ΔR²** |
| --- | --- | --- | --- | --- | --- |
| lag-0 (primary) | 1.0037 (0.007) | 1.0033 (0.039) | 1.0032 (0.020) | 1.0069 (<0.001) | +0.005 |
| lag-1 | 1.0038 (0.007) | 1.0017 (0.287) | 1.0029 (0.041) | 1.0058 (<0.001) | +0.004 |
| lag-2 | 1.0046 (0.001) | 1.0010 (0.510) | 1.0013 (0.349) | 1.0028 (0.062) | +0.003 |
| lag-3 | 1.0035 (0.016) | 0.9993 (0.666) | 1.0001 (0.947) | 1.0014 (0.348) | -0.006 |
| Mean, lags 0–2 | 1.0057 (0.001) | 1.0030 (0.120) | 1.0035 (0.035) | 1.0077 (<0.001) | +0.008 |
| Mean, lags 0–3 | 1.0063 (<0.001) | 1.0023 (0.273) | 1.0030 (0.092) | 1.0073 (<0.001) | +0.006 |

*IRR > 1 indicates higher attendance with higher pressure. The association attenuated at longer individual lags (significant only in FY2022 at lag-3), and no exposure produced a stable out-of-sample gain (mean ΔR² ≤ +0.008).*
