# Supplementary material for: Evaluating a candidate predictor beyond statistical significance: Atmospheric pressure and psychiatric day-care attendance as a worked example
Source: PLoS One. 2026 Nov 6;21(11):e0352385. doi: 10.1371/journal.pone.0352385 (PMC13742932; doi:10.1371/journal.pone.0352385)
Supplement: Supplementary file 3 [file pone.0352385.s005.docx]

**S3 Table. Sensitivity to the lag-14 attendance covariate.**

Same-day atmospheric pressure incidence rate ratio (IRR, per hPa) estimated with the primary model (weekday indicators plus lag-14 same-weekday attendance) and with the lag-14 term removed (weekday indicators only), on the same analytic sample. *p*-values are shown for completeness.

| **FY** | **Primary model (weekday + lag-14) IRR [95% CI] (p)** | **Without lag-14 (weekday only) IRR [95% CI] (p)** |
| --- | --- | --- |
| 2022 | 1.0037 [1.0010, 1.0065] (0.007) | 1.0070 [1.0040, 1.0099] (<0.001) |
| 2023 | 1.0033 [1.0002, 1.0064] (0.039) | 1.0028 [0.9997, 1.0059] (0.079) |
| 2024 | 1.0032 [1.0005, 1.0059] (0.020) | 1.0038 [1.0011, 1.0066] (0.006) |
| 2025 | 1.0069 [1.0040, 1.0098] (<0.001) | 1.0085 [1.0055, 1.0116] (<0.001) |

Forward-validation predictive performance with the lag-14 term removed (weekday-only baseline), five-fold expanding-window cross-validation:

| **FY** | **Baseline R²** | **+ Pressure R²** | **ΔR²** |
| --- | --- | --- | --- |
| 2022 | 0.518 | 0.552 | +0.033 |
| 2023 | 0.597 | 0.591 | −0.006 |
| 2024 | 0.535 | 0.521 | −0.015 |
| 2025 | 0.628 | 0.677 | +0.049 |

*Note.* Removing the lag-14 term leaves the same-day pressure IRR essentially unchanged in direction and magnitude (it does not increase systematically across years), indicating that the association is neither created nor strongly masked by the lag-14 covariate; the conclusions are therefore not an artifact of including it. With the lower weekday-only baseline, adding pressure still produces small, sign-inconsistent changes in out-of-sample R² (negative in two of four years), so the absence of a stable predictive gain does not depend on the lag-14 specification. Interpretation focuses on effect sizes and intervals rather than on significance thresholds.
