# Supplementary material for: Evaluating a candidate predictor beyond statistical significance: Atmospheric pressure and psychiatric day-care attendance as a worked example
Source: PLoS One. 2026 Nov 6;21(11):e0352385. doi: 10.1371/journal.pone.0352385 (PMC13742932; doi:10.1371/journal.pone.0352385)
Supplement: Supplementary file 4 [file pone.0352385.s007.docx]

Response to the Academic Editor and Reviewers

**Manuscript ID: PONE-D-26-28046**

Title: Evaluating a candidate predictor beyond statistical significance: Atmospheric pressure and psychiatric day-care attendance as a worked example

Dear Dr. Yao and Reviewers,

We sincerely thank the Academic Editor and the three reviewers for their careful and constructive evaluation of our manuscript. We have substantially reorganized the manuscript in response to the comments. The principal change is a recentering of the paper from the specific atmospheric-pressure finding to the broader methodological process of evaluating a candidate predictor through three distinct questions: statistical detectability, robustness of the estimated association to reasonable adjustment and alternative specifications, and incremental out-of-sample predictive utility. Atmospheric pressure is now presented explicitly as a pre-specified worked example rather than as the principal substantive contribution of the study.

# Major changes

- The title and short title have been revised to foreground the candidate-predictor evaluation framework rather than the specific atmospheric-pressure finding.
- The Abstract has been rewritten to foreground the three-part evaluation framework, clarify the distinct roles of atmospheric pressure and the other meteorological variables, and describe the predictive baseline accurately as incorporating scheduling variables and recent attendance history.
- The Introduction has been substantially reorganized around candidate-predictor evaluation rather than atmospheric pressure itself.
- A new Related work section has been added, covering meteorological factors and psychiatric service use, calendar and weather variables in health-service forecasting, and the distinction between association, robustness, and prediction.
- The Materials and methods and Results have been reorganized around detectability, effect-estimate robustness, and predictive utility. Robustness is evaluated primarily from stability of direction, magnitude, and precision rather than binary persistence of p < 0.05. Expanding-window cross-validation is identified as the primary temporally ordered validation, whereas repeated K-fold cross-validation is treated as a secondary non-temporal resampling sensitivity analysis. Because the candidate predictor is same-day daily mean pressure, the manuscript describes predictive utility rather than prospective operational forecasting.
- The exposure definition has been clarified: the pre-specified exposure was the same-day mean atmospheric-pressure level, not within-day or day-to-day pressure change. The manuscript therefore does not claim to test effects of transient barometric changes.
- Tables 3a and 3b now report 95% confidence intervals together with IRRs and p-values for the robustness analyses. The associated Results text has been synchronized to the higher-precision p-values, and cross-year robustness is described by the pattern of effect-estimate stability rather than by a simple count of statistically significant years.
- The Discussion has been restructured so that methodological implications precede the substantive interpretation of atmospheric pressure, and the pressure-specific interpretation has been made more cautious.
- The discussion of FY2025, multiplicity, setting dependence, and generalizability has been expanded, including an illustrative probability calculation requested by Reviewer #2 and an explicit distinction between the binary significance pattern and robustness of the effect estimate.
- Variance-inflation diagnostics have been added to address Reviewer #1’s concern regarding multicollinearity. Across all meteorological and seasonal adjustment models, the pressure VIF was 1.05–1.65 and other conventional adjustment-term VIFs were ≤2.6.
- The Limitations now explicitly state that other potentially relevant meteorological dimensions, such as sunshine duration and wind conditions, were not evaluated.
- The journal’s formatting requirements have also been incorporated: the ethics statement appears only in Materials and methods; manuscript keywords and section numbering have been removed; the former Declarations block has been removed with Acknowledgments retained as a standalone section; and the generative-AI disclosure is placed in Materials and methods.

# Academic Editor

## Comment (verbatim)

*This manuscript has a valuable core message, but it is currently "hidden" behind a framing that overemphasize a single weather variable (atmospheric pressure) while the real contribution is the methodological framework for evaluating any candidate predictor.*

*Please treat this revision as an opportunity to recenter the narrative around the process of model evaluation (detectability--robust--utility) rather than the specific finding on pressure. We expect the revised manuscript to be substantially reorganized, with a clearer abstract, a new related-work section, and a more cautious discussion.*

## Response

We thank the Academic Editor for identifying this central framing issue. We agree that the previous version placed too much narrative emphasis on atmospheric pressure even though the intended contribution was methodological. We have therefore substantially reorganized the manuscript around a three-part evaluation of a pre-specified candidate predictor: statistical detectability, robustness of the estimated association to reasonable adjustment and alternative specifications, and incremental out-of-sample predictive utility. Robustness is now described in terms of stability of direction, magnitude, and precision rather than whether p-values remain below 0.05. Tables 3a and 3b now display 95% confidence intervals for the robustness analyses so that this stability can be evaluated directly. The primary predictive assessment preserves temporal ordering through expanding-window cross-validation; repeated K-fold analyses are explicitly described as secondary non-temporal sensitivity analyses. Because same-day daily mean pressure is not fully observed until the day is complete, the manuscript also avoids presenting the analysis as prospective operational forecasting. The Abstract, Introduction, Materials and methods, Results, Discussion, and Conclusion have been revised accordingly, and a new Related work section has been added. The revised manuscript now makes explicit that its purpose is not to establish a general biometeorological effect of atmospheric pressure, but to illustrate how a statistically detectable candidate predictor may show specification-sensitive effect estimates and little incremental out-of-sample predictive value. **Changes:** Title and Abstract (pp. 1–2, lines 1–35); Introduction (pp. 2–4, lines 36–81); Related work (pp. 4–6, lines 82–142); Materials and methods (pp. 7–13, lines 143–307); Results (pp. 14–21, lines 308–459); Discussion (pp. 21–28, lines 460–644); Conclusion (pp. 28–29, lines 645–663).

# Journal Requirements

## Requirement 1: PLOS ONE style and file naming

Thank you. We have reformatted the revised manuscript against the supplied PLOS ONE templates. The manuscript now uses the PLOS-style heading hierarchy without section numbering, omits a manuscript Keywords line, places tables and the figure caption after their first citation, uses continuous line numbering and page numbering, and removes the former Declarations block. Acknowledgments is retained as a standalone section, while the generative-AI disclosure appears in Materials and methods. The corresponding-author format, reference formatting, and supporting-information captions have also been aligned with the supplied templates. In addition, all figures were checked against the PLOS ONE figure guidelines, and the figure files were prepared to meet the specified requirements for file format, resolution, and dimensions. **Changes:** Formatting throughout the manuscript (pp. 1–34).

## Requirement 2: Ethics statement should appear only in the Methods

Thank you. We have retained the complete ethics statement in Materials and methods and removed the duplicate ethics statement from all other parts of the manuscript. **Changes:** Materials and methods, Study design and setting (p. 7, lines 154–164).

## Requirement 3: Reviewer-suggested references

Thank you. No reviewer required citation of a specific publication. In response to the request for a more complete account of related work, however, we reviewed relevant literature on health-service forecasting and the distinction between explanatory association and predictive evaluation and added the publications that we considered directly relevant to the revised framing. **Changes:** Related work (pp. 4–6, lines 82–142).

# Reviewer #1

## Comment (verbatim)

*This study offers a critical methodological lesson in observational research, demonstrating that statistical significance does not equal a robust or practically useful effect. Although a statistically significant association between atmospheric pressure and daily attendance was detected across four fiscal years in a Japanese psychiatric day-care center, this link failed to remain robust when adjusting for confounding weather factors (such as precipitation, which directly reduced attendance) and provided negligible out-of-sample predictive improvement beyond the fixed schedule, which alone explained 55%–69% of the variance. While methodologically sound, the study exhibits notable scientific limitations, including a single-center design with a small cohort, aggregation of diverse psychiatric diagnoses, multicollinearity among weather variables, and meteorological data collected 20 km away; consequently, while these data effectively refute the predictive utility of weather for this specific facility, they remain insufficient to rule out general biometeorological influences on mental health.*

## Response

**Single-facility design and study scale.** We thank the reviewer for this careful assessment and agree with these limitations. We also acknowledge directly the single-facility design and limited scale of the study. Each fiscal-year analysis included 262–266 analytic operating days, and the FY2025 registered cohort was modest (N = 59); these features limit the precision and generalizability of the year-specific estimates.

**Diagnostic heterogeneity and ecological outcome.** The facility-level ecological outcome cannot identify weather sensitivity within diagnostic, demographic, or transport-defined subgroups.

**Multicollinearity.** We addressed the concern regarding multicollinearity quantitatively. Variance inflation factors were calculated in the meteorological and seasonal adjustment models using the same design matrices as the fitted models. Across all such models, the VIF for atmospheric pressure ranged from 1.05 to 1.65, and VIFs for the other meteorological or seasonal adjustment terms were ≤2.6 where conventional term-wise VIFs were defined. In the natural cubic spline models, the pressure VIF was likewise low (1.24–1.41); individual spline-basis VIFs were not interpreted because of their structural dependence with the intercept under this coding. These diagnostics provide no evidence that the marked attenuation of the pressure estimate, including the movement of the FY2022 estimate toward the null after seasonal adjustment, resulted from problematic variance inflation or severe multicollinearity. We nevertheless retain a cautious interpretation because low VIFs do not exclude shared meteorological or seasonal structure, measurement error, or confounding as possible explanations for attenuation. The revised manuscript further clarifies that robustness is judged primarily from stability of effect direction, magnitude, and precision rather than whether individual p-values cross 0.05. Tables 3a and 3b now report 95% confidence intervals for all displayed robustness estimates.

**Meteorological exposure measurement.** The limitation concerning meteorological measurements from representative observation stations approximately 20 km away is retained. We also state that other potentially relevant meteorological dimensions, such as sunshine duration and wind conditions, were not evaluated.

**Scope of generalization.** We have strengthened the Discussion and Limitations to emphasize that the findings concern aggregate attendance at one highly schedule-driven psychiatric day-care and do not demonstrate an absence of weather sensitivity at the individual level or in psychiatric populations generally. We also revised the Abstract and Conclusion so that the substantive conclusion is explicitly setting-specific and does not imply that weather lacks relevance to psychiatric service utilization more broadly. Setting dependence, including fixed scheduling and relatively frequent private-car use, is discussed explicitly.

**Changes:** Abstract (pp. 1–2, lines 9–35); Materials and methods, Robustness to adjustment and alternative specifications (pp. 10–12, lines 223–258); Results, Robustness (pp. 15–18, lines 342–404); Discussion, Interpreting the atmospheric-pressure example (pp. 23–25, lines 517–576); Setting dependence and generalizability (pp. 25–26, lines 577–596); Limitations (pp. 26–28, lines 597–644); Conclusion (pp. 28–29, lines 645–663).

# Reviewer #2

## General comment (verbatim)

*This manuscript presents a retrospective, facility-level observational study examining the association between same-day atmospheric pressure and daily attendance at a psychiatric day-care unit in Japan over four fiscal years. The authors utilize quasi-Poisson regression and cross-validation to separate three distinct properties of the reported association: statistical detectability, robustness to confounding, and predictive utility. The study finds that while atmospheric pressure is a statistically significant correlate of attendance in all four years, the association is generally not robust to adjustment for other meteorological variables and provides negligible improvement in out-of-sample prediction beyond the fixed institutional schedule. However, there are several areas where the manuscript could be strengthened.*

## Response

We thank the reviewer for this accurate summary and for the two specific suggestions below. We have addressed both by expanding the discussion of multiplicity and chance and by substantially strengthening the setting-specific generalizability discussion. We have also refined the robustness language throughout the revised manuscript so that it is based on stability of direction, magnitude, and precision rather than a binary count of whether adjusted p-values remain below 0.05.

## Comment 1 (verbatim)

*1 The authors highlight FY2025 as the only year where the pressure association survived all confounding adjustments. To strengthen this section, the authors should explicitly calculate or discuss the statistical probability of finding one “surviving” year out of four purely by chance, given the number of adjustments tested.*

## Response

We thank the reviewer for this important point. We agree that the FY2025 pattern requires explicit consideration of multiplicity and chance. We have therefore added an illustrative conservative calculation for the binary significance pattern. Under a global null in which each individual test is calibrated at alpha = 0.05, the probability that a given year remains statistically significant under every adjustment cannot exceed 0.05. If the four fiscal years are additionally treated as independent for illustration, using this upper bound gives a probability of exactly one surviving year of 4(0.05)(0.95)^3 = 0.171 and a probability of at least one surviving year of 1 - (0.95)^4 = 0.185. We explicitly note that these values are illustrative benchmarks rather than formal probabilities because the adjustment models share the same observations and most covariates and the fiscal years cannot be assumed to be strictly exchangeable or independent. A precise null probability would require a resampling or simulation procedure reproducing the complete analysis pipeline. Importantly, the revised manuscript states that this calculation pertains only to the binary significance pattern and is not the criterion used to define robustness; robustness is evaluated from stability of effect direction, magnitude, and precision. We therefore continue to regard the FY2025 finding as weak and exploratory rather than as evidence of a reproducible year-specific effect. **Changes:** Discussion, Interpreting the atmospheric-pressure example (pp. 24–25, lines 549–568); Limitations (pp. 26–27, lines 606–611).

## Comment 2 (verbatim)

*2 The discussion on generalizability could be expanded. The setting described (patients commuting by private car, high attendance adherence to a fixed schedule) might be specific to this Japanese suburban context.*

## Response

We agree and have expanded this discussion substantially. A subsection, “Setting dependence and generalizability,” now explicitly relates the limited incremental contribution of weather to pre-assigned weekdays, the biweekly program rotation, recent attendance history, and relatively frequent use of private cars. We also clarify that weather could have greater predictive relevance in services where attendance is more discretionary, scheduling is flexible, or patients depend more heavily on walking or public transportation, and that acute or emergency psychiatric services may differ still further because presentation is more symptom-driven. The revised manuscript therefore characterizes the present facility as representing the structure-dominated end of a continuum rather than implying that weather lacks relevance to psychiatric service utilization generally. **Changes:** Discussion, Setting dependence and generalizability (pp. 25–26, lines 577–596); Conclusion (pp. 28–29, lines 645–663).

# Reviewer #3

## Comment 1 (verbatim)

*The paper in my opinion does not demonstrate any significant findings apart from some unstable when adjusting significance of air pressure and daily attendance. Many weather factors are excluded, i.e. when you speak about weather variables you cannot speak only about air pressure. Initial the paper gives the impression, and states it in some parts that takes into account only the pressure and performs exploratory research. But then in other parts the authors mentions that finally they considered also additional variables.*

## Response

**Principal empirical finding.** We thank the reviewer for this comment. We agree that the atmospheric-pressure results should not be presented as evidence of a strong or generalizable biometeorological effect. We respectfully clarify that the principal empirical finding is not the presence of a large atmospheric-pressure effect. Rather, the study shows that a pre-specified candidate predictor can be repeatedly statistically detectable while its effect estimates remain sensitive in magnitude or precision to reasonable adjustment and while it adds little stable incremental out-of-sample predictive utility beyond a strong structural baseline. We regard this divergence across detectability, robustness, and predictive utility—not a pressure-specific biometeorological effect—as the interpretable finding illustrated by the data. The robustness results are now reported with IRRs and 95% confidence intervals in Tables 3a and 3b and described by their year-specific effect-estimate stability: marked attenuation in FY2022, moderate attenuation after precipitation adjustment in FY2023, specification-sensitive precision in FY2024, and comparatively greater stability in FY2025. This avoids defining robustness by a simple significant/non-significant classification.

**Scope of meteorological variables.** The revised paper therefore treats atmospheric pressure as one pre-specified candidate predictor used as a worked example, not as a claim that pressure is the only relevant meteorological dimension. Finally, the Limitations now state that other potentially relevant meteorological dimensions, such as sunshine duration and wind conditions, were not evaluated. These changes delimit the scope of the study without implying that the meteorological variables examined exhaust the concept of “weather.”

**Role and selection of meteorological variables.** We also agree that the distinct roles of the meteorological variables needed to be made explicit. Temperature, relative humidity, and precipitation are now described consistently from the Abstract onward as pre-planned co-varying meteorological factors used to stress-test the robustness of the pressure estimate rather than as additional candidate predictors selected after inspection of the results. No data-driven selection among these weather variables was performed.

**Exposure definition.** The revised manuscript also explicitly states that the pre-specified exposure was the same-day mean atmospheric-pressure level rather than within-day or day-to-day pressure change and therefore does not directly test transient barometric changes.

**Reproducibility of the statistical analysis.** To facilitate independent assessment of the statistical analysis, the revised Materials and methods also state the rationale for quasi-Poisson estimation for the underdispersed daily counts and its dispersion-based inference, report the software and package versions used, and provide the anonymized analytic dataset and analysis code reproducing the reported results in the public Zenodo repository (DOI: 10.5281/zenodo.20518605).

**Changes:** Abstract (pp. 1–2, lines 9–35); Introduction (pp. 2–4, lines 36–81); Materials and methods, Meteorological data and candidate predictor (pp. 8–9, lines 176–196) and Software and reproducibility (p. 13, lines 295–302); Results, Robustness (pp. 15–18, lines 342–404); Limitations (pp. 26–28, lines 597–644); Conclusion (pp. 28–29, lines 645–663).

## Comment 2 (verbatim)

*Similar work respecting the idea and similar work using the same techniques and methods is missing and that is something vital even for this journal.*

## Response

We agree and have added a new Related work section organized into three parts: meteorological factors and psychiatric service use; calendar and weather variables in health-service forecasting; and the distinction between statistical association, robustness, and predictive utility. In addition to reorganizing relevant references from the previous Introduction, we now discuss specific prior studies that are directly relevant to the revised framing. These include Luo et al. [21] on time-series forecasting of outpatient visits, Bai et al. [22] on outpatient-visit forecasting using meteorological information, and studies evaluating calendar and weather predictors in emergency-department demand forecasting [23-25]. In particular, Erkamp et al. [25] directly compared calendar-only and calendar-plus-weather models, providing a close precedent for our evaluation of whether meteorological information adds predictive value beyond a strong structural baseline. We also expanded the methodological context by discussing Shmueli [1] on explanatory versus predictive modeling, together with work distinguishing predictor from explanatory-factor questions and assessing the incremental value of candidate predictors [26,27]. These additions place the present study more explicitly within both the health-service forecasting literature and the methodological literature relevant to the detectability–robustness–predictive-utility distinction. **Changes:** Related work, particularly Calendar and weather variables in health-service forecasting and From association to robustness and predictive utility (pp. 5–6, lines 101–142).

## Comment 3 (verbatim)

*So the authors should re-write the whole paper, adding related work, explaining clearly which variables the used, in abstract and in the text.*

## Response

We agree. The manuscript has been substantially reorganized rather than revised only locally. The Abstract now states the three-stage evaluation explicitly and distinguishes the pre-specified candidate predictor from the co-varying meteorological factors. The Introduction explains why atmospheric pressure is used as a worked example, and the Materials and methods define the roles of atmospheric pressure, temperature, relative humidity, precipitation, seasonality, the scheduling variables, and recent attendance history before the analyses are presented. The Abstract also now describes the predictive baseline accurately as incorporating both scheduling variables and recent attendance history. The Results follow the same sequence—detectability, effect-estimate robustness, and predictive utility—and the Discussion interprets these components separately. **Changes:** Abstract (pp. 1–2, lines 9–35); Introduction (pp. 2–4, lines 36–81); Related work (pp. 4–6, lines 82–142); Materials and methods (pp. 7–13, lines 143–307); Results (pp. 14–21, lines 308–459); Discussion (pp. 21–28, lines 460–644); Conclusion (pp. 28–29, lines 645–663).

## Comment 4 (verbatim)

*Then they should focus on appropriate structure.*

## Response

We agree. The revised manuscript uses unnumbered PLOS-style hierarchical headings and a structure centered on the methodological framework: Introduction—the general problem of distinguishing detectability, robustness, and predictive utility; Related work—psychiatric meteorological studies, health-service forecasting, and methodological context; Materials and methods—the three-stage evaluation framework and explicit roles of the candidate predictor, meteorological covariates, structural baseline, primary temporally ordered validation, and secondary non-temporal sensitivity analyses; Results—detectability, effect-estimate robustness, and predictive utility reported sequentially; Discussion—methodological implications first, followed by cautious interpretation of atmospheric pressure, generalizability, and limitations; and Conclusion—a concise statement of the setting-specific result and the broader methodological lesson. **Changes:** Throughout the main manuscript (pp. 1–29, lines 1–663).

We again thank the Academic Editor and Reviewers for their constructive comments. We believe that the substantial reorganization and clarification prompted by the review have strengthened the manuscript and made its methodological contribution considerably clearer.
