# Supplementary material for: Evaluating a candidate predictor beyond statistical significance: Atmospheric pressure and psychiatric day-care attendance as a worked example
Source: PLoS One. 2026 Nov 6;21(11):e0352385. doi: 10.1371/journal.pone.0352385 (PMC13742932; doi:10.1371/journal.pone.0352385)
Supplement: Supplementary file 5 [file pone.0352385.s008.docx]

Response to the Academic Editor and Reviewers

Manuscript ID: PONE-D-26-28046R1

Title: Evaluating a candidate predictor beyond statistical significance: Atmospheric pressure and psychiatric day-care attendance as a worked example

Dear Dr. Yao and Reviewers,

We thank the Academic Editor and both reviewers for this further assessment, and we are grateful to Reviewer #2 for the generous comment on the previous revision. Both remaining suggestions have been implemented, and the reference list has been reviewed as the journal requested. The changes in this revision are confined to one new figure, one new Discussion subsection, the consequent figure renumbering, and three small corrections to the reference list. No analysis was repeated, no data were added, and no reported result has changed.

# Summary of changes

- A new Fig 1 presents the three-stage framework for evaluating a candidate predictor (p. 9, lines 203–204 for the citation; p. 10, lines 205–209 for the caption).
- The figure previously numbered Fig 1 (residual autocorrelation diagnostics) is now Fig 2; its citation and caption have been updated (p. 21, line 447 for the citation; lines 454–456 for the caption). The figure file was also re-rendered at its final published size so that its axis labels fall within the 8–12 point range required by the figure guidelines; the plotted content is unchanged. S1 Fig and S2 Fig are unchanged.
- A new Discussion subsection, "Applications of the framework", has been added before the Limitations (p. 27, lines 603–627).
- Three minor inaccuracies in the reference list have been corrected (p. 33, line 741; p. 34, lines 772 and 784).

# Journal requirements

## Review of the reference list, including retracted articles

We have reviewed the complete reference list. Each of the 39 entries was checked against the publisher record, and retraction status was verified against the Retraction Watch database as indexed in OpenAlex, supplemented by a targeted search for each clinical and empirical reference. No cited article is retracted, and none carries a correction or an expression of concern; no retracted work therefore needs to be flagged, and no retraction notice needs to be cited.

The review did identify three minor inaccuracies, which we have corrected. In reference 17 the opening words of the published title ("A study of the") had been omitted. In reference 28 the STROBE title was truncated and now reads in full, "…(STROBE) statement: guidelines for reporting observational studies." Reference 33 was the only entry without a DOI, and the DOI (10.1093/biomet/37.3-4.409) has been added. No references were added or removed, and the numbering is unchanged, so no in-text citation was affected. **Changes:** References (p. 33, line 741; p. 34, lines 772 and 784).

# Reviewer #1

## Comment

All comments have been addressed; no further comments were provided.

## Response

We thank the reviewer for the careful assessment of both the original submission and the previous revision, which substantially improved the manuscript.

# Reviewer #2

## Comment 1

"A figure of the overall research framework can be added to improve the readability."

## Response

We agree, and have added Fig 1, which sets out the three-stage evaluation: detectability, robustness, and incremental predictive utility. Each stage is shown with the question it answers, how it is assessed, and what it does not establish, so that the distinction at the center of the paper can be grasped at a glance rather than reconstructed from the text.

Two design choices are worth noting. First, the figure is deliberately generic: it describes the sequence for any pre-specified candidate predictor rather than restating the atmospheric-pressure results. This keeps it consistent with the Academic Editor's guidance that the contribution of the paper is the evaluation framework rather than the particular finding, and it also speaks to the reviewer's second comment, since a reader can see directly how the sequence would transfer to another predictor. Second, the caption maps the generic elements onto the present study and onto the corresponding tables, so the figure doubles as a guide to the Results, which are organized in the same three stages.

Because the new figure is first cited in the Materials and methods, it precedes the residual diagnostics figure in the text. Following the convention that figures are numbered in order of first citation, the diagnostics figure is now Fig 2, and its in-text citation and caption have been updated. In preparing the renumbered file we noticed that this figure had been drawn on a larger canvas and then stored at its final dimensions, with the result that its axis labels rendered at roughly six points rather than the 8–12 points required by the figure guidelines. We have re-rendered it at its final published size using the same data and the same model specification; the plotted values, panel arrangement, and colors are unchanged, and the axis labels are now legible at the published size. The supporting figures are unaffected. **Changes:** Materials and methods, Statistical evaluation framework (p. 9, lines 203–204); Fig 1 caption (p. 10, lines 205–209); Results, Model diagnostics and cross-year consistency (p. 21, line 447); Fig 2 caption (p. 21, lines 454–456).

## Comment 2

"Some applications of the proposed method can be discussed."

## Response

We have added a new Discussion subsection, "Applications of the framework", placed after "Setting dependence and generalizability" and before the Limitations. It identifies settings in which the same sequence applies: health-service demand forecasting, where candidate external predictors such as weather, air quality, public holidays, local events, or internet search activity are regularly proposed as improvements to operational forecasts; clinical prediction modeling, where incremental value beyond the predictors already in a model is the accepted criterion for adding a new marker [27]; and signals derived from wearable devices and smartphone use in mental health research, where a statistically significant association with a routinely collected outcome is often followed quickly by an operational claim.

The subsection also makes two practical points that we hope will help readers apply the sequence. The three stages require no specialized methodology and cost little beyond the model that would be fitted in any case. More importantly, what the third stage can show depends on how the baseline is specified: a baseline containing only the candidate predictor will almost always suggest an apparent gain, whereas a baseline containing the information the setting already holds asks the question that matters operationally. We therefore recommend that the baseline be reported explicitly, so that readers can judge whether a small increment reflects a weak predictor or an already strong baseline. **Changes:** Discussion, Applications of the framework (p. 27, lines 603–627).

We thank the Academic Editor and the reviewers once again. We hope that the revised manuscript is now suitable for publication in PLOS ONE.
